# The microbiome of Crohn’s disease aphthous ulcers

**DOI:** 10.1186/s13099-018-0265-6

**Published:** 2018-10-10

**Authors:** Claire L. O’Brien, Christopher J. Kiely, Paul Pavli

**Affiliations:** 1 Medical School, Australian National University, Canberra, ACT 2600 Australia; 2 Gastroenterology and Hepatology Unit, Canberra Hospital, Lvl 5, Bldg 10, Canberra Hospital, Yamba Drive, Garran, Canberra, ACT 2605 Australia

**Keywords:** Crohn disease, Microbiome, Inflammatory bowel disease

## Abstract

**Background:**

Reduced intestinal microbial diversity and bacterial imbalance (dysbiosis) are seen in studies of Crohn’s disease. As it is difficult to obtain biopsy samples before disease presentation, the earliest mucosal lesions in Crohn’s disease, aphthous ulcers, present the best chance at assessing microbial communities at the onset of disease or a new flare. The aim of our study was to compare the microbial communities of aphthous ulcers and adjacent normal mucosa from patients with Crohn’s disease with normal mucosa from controls.

**Results:**

We did not observe bacterial imbalance or reduced alpha diversity in Crohn’s disease aphthous ulcers and adjacent mucosa, relative to control biopsies. *Bacteroides* were common to all Crohn’s disease and control samples, and there were no bacterial taxa unique to aphthous ulcers. The relative abundance of *Faecalibacterium* was not reduced in aphthous ulcers relative to control mucosa, and was not more likely to be detected in control samples.

**Conclusions:**

In contrast to well-documented changes seen in late-stage Crohn’s disease, microbial communities of aphthous ulcers do not display evidence of bacterial imbalance or reduced diversity. Our data suggest that dysbiosis occurs during active disease, and improves when patients are in remission.

## Background

Crohn’s disease (Crohn’s disease), a chronic, relapsing inflammatory disease of the gastrointestinal tract, is thought to result from an aberrant, ongoing immune response to bacteria, in genetically susceptible individuals. Over 200 gene variants are associated with IBD, with just over 30 of these being Crohn’s disease-specific [1]. Analysis of these gene variants suggests that host–microbe interactions are crucial in the development of Crohn’s disease.

Several lines of evidence suggest that microbes play a role in either the onset or perpetuation of Crohn’s disease. The earliest lesion in Crohn’s disease is the aphthous ulcer, which overlies Peyer’s patches in the small bowel, and lymphoid follicles in the large bowel. These lymphoid aggregates are the site of luminal antigen sampling by innate immune cells. Granulomas, which are a histological hallmark of Crohn’s disease, contain bacteria [2]. Temporary diversion of the fecal stream to a proximal ileostomy prevents the recurrence of inflammation in down-stream mucosal sites [3]. Numerous studies have shown that the gut microbiome is dysregulated in Crohn’s disease, both in terms of its species composition and its function [4–6].

Reduced alpha diversity (mean number of bacterial species in a given sample) is frequently observed in the microbial communities of Crohn’s disease mucosa when compared to mucosa from healthy controls, and cannot be attributed to inter-individual variation in the gut microbiome [7]. Studies consistently show that the gut microbiome of Crohn’s disease patients is depauperate, in particular butyrate-producing *Faecalibacterium prausnitzii* and *Roseburia* [8–11]. Other groups of bacteria, such as Enterobacteriaceae, which includes *Escherichia coli*, are increased in Crohn’s disease relative to controls [4, 12–14], and their abundance has been shown to correlate with disease severity [4]. Although a number of bacterial taxa have been implicated in Crohn’s disease, no single causative organism has been identified.

There is tremendous inter-individual variation in the gut microbiome of healthy individuals; however, despite this, the functional capacity of each individual’s microbiome remains similar [7]. Conversely, modest differences in the taxonomic composition of the gut microbiome of patients with IBD are associated with major changes in its function [15]. These changes may reflect the response of bacteria to an inflamed gut, as enrichment in microbial pathways that enable bacteria to cope with oxidative stress, evade immune responses, and take up host metabolites without prior synthesis (auxotrophy) is observed. There are also corresponding reductions in short chain fatty acid (SCFA) and amino acid biosynthesis, as well as gut carbohydrate metabolism [15]. Proving that changes in the gut microbiome precede the onset of disease, cause a disease flare, or are a consequence of inflammation remains challenging.

It is almost impossible to obtain mucosal samples from people prior to the development of IBD, and only a few studies have assessed the mucosal microbiome of patients with Crohn’s disease at the onset of disease. A large study of paediatric patients with new-onset Crohn’s disease showed that species-richness was reduced in Crohn’s disease, and that the abundance of several taxa was altered [4]. However, an inflammatory response was well established in 96% of the patients recruited to this study [mild (PCDAI 10–30)—moderate/severe disease (PCDAI > 30)]. Dysbiosis is associated with other inflammatory conditions, such as obesity [16] and Type 2 diabetes [17], suggesting that chronic inflammation drives changes in the gut microbiome. A study by Kiely et al. showed that the mucosal microbiome of patients with inflammatory bowel disease (IBD) fluctuates over time, with greater changes observed in patients who had ongoing microscopic inflammation [18]. The majority of studies support the idea that dysbiosis is a common response to chronic inflammation.

The earliest mucosal lesions in Crohn’s disease, aphthous ulcers, are small (1–5 mm) superficial ulcerations surrounded by a ring of erythema then normal surrounding mucosa [19]. These lesions overlie the follicle associated epithelium (FAE) of the small bowel (Peyer’s patches) and large bowel (lymphoid follicles) [20], can be found in 70% of patients with Crohn’s disease [21], appear more commonly in the distal ileum [19, 21], and can develop into larger, transverse linear ulcers [19]. Approximately 10% of the epithelial cells of the FAE are microfold cells, commonly referred to as ‘M’ cells. These cells have a reduced glycocalyx and blunted microvilli, and are highly specialized in phagocytosis and transcytosis of luminal antigens, which they package in vesicles and deliver to underlying immune cells [22].

Numerous bacterial pathogens, including *Yersinia pseudotuberculosis* [23], *Mycobacterium tuberculosis* [24], *Salmonella typhimurium* [25], *Shigella flexneri* [26], and *Escherichia coli* [27, 28] exploit M cells to cross the epithelial barrier and cause infection. Adherent invasive *E. coli* (AIEC), which have been implicated in Crohn’s disease, target M cells on Peyer’s patches through the expression of one of two major long polar fimbriae (*lpfA*) operons, which allows them to translocate the intestinal epithelial barrier [29]. However not all AIEC strains harbor *lpfA* [30], and non-AIEC strains may harbor *lpfA*, suggesting that this is not the only mechanism by which AIEC exploit M cells of the FAE.

Because of the difficulty in obtaining biopsy samples before Crohn’s disease presentation, aphthous ulcers represent the earliest stage at which microbial communities can be assessed at the onset of disease or a disease flare [31]. The aim of our study was to compare the microbial communities of aphthous ulcers and adjacent mucosa from individuals with Crohn’s disease with mucosa from healthy controls, to determine whether or not specific bacteria, or an imbalance in the gut microbiome, are present in the initial Crohn’s disease lesion. This is the first study to assess the bacterial community composition of aphthous ulcers in Crohn’s disease.

## Results

### Subject characteristics

The clinical characteristics of the 29 patients who underwent colonoscopy are summarised in Table 1. Two patients were on antibiotics at the time of the procedure (Patients 6 and 12 with Crohn’s disease). The average time from diagnosis of IBD was 8.9 years (range 0–25 years).

**Table 1:** Patient and sample characteristics

Sample ID	Age (years)	Gender	Disease status	Biopsy location	Years since diagnosis	Montreal location	Montreal behaviour	Antibiotics	Indication for colonoscopy (controls)
1SBAU	40	M	CD	TI ulcer	8	L1	B1	N	
1SB				CM					
2SBAU	29	F	CD	TI ulcer	7	L3	B1	N	
2SB				TI					
3LBAU	37	M	CD	SIG ulcer	18	L2	B1	N	
3LB				AC					
4SBAU	32	M	CD	TI ulcer	3	L3	B2p	N	
4SB				TI					
5LBAU	30	M	CD	SIG ulcer	6	L3	B1	N	
5LB				SIG					
6RECAU	50	F	CD	REC ulcer	0	L2	B1	Y	
6REC				REC					
7LBAU	40	M	UC	SIG ulcer	25	E3	S2	N	
7LB				DC					
8SBAU	34	F	CD	CM ulcer	15	L3	B2	N	
8SB				ICV					
9SBAU	22	F	CD	CM ulcer	0	L3	B1	N	
9SB				CM					
10RECAU	22	F	CD	REC ulcer	0	L3	B1	N	
10REC				REC					
11SBAU	50	M	CD	TI ulcer	10	L3	B3p	N	
11SB			CD	TI					
12LBAU	28	M	CD	SIG ulcer	15	L3	B2	Y	
12LB				DC					
NB1	58	M	NC	DC	–				Rectal bleeding
NB2	31	F	NC	AC	–				Constipation
NB5	55		NC	TI	–				Altered bowel habit
NB11	33	M	NC	TI	–				Diarrhoea
NB12	38	M	NC	TI	–				Rectal bleeding
NB13	35	M	NC	REC	–				Rectal bleeding
NB15	57	M	NC	TI	–				Rectal bleeding, abdominal pain
NB17	43	M	NC	TI	–				Rectal bleeding
NB18	34	M	NC	TI	–				Rectal bleeding, FHCRC
NB21	53	F	NC	TI	–				Bloating, abdominal pain
NB23	43	M	NC	TI	–				FHCRC
NB26	60	F	NC	SIG	–				Rectal bleeding
NB27	58	M	NC	TI	–				Polyp surveillance
NB29	37	F	NC	TI	–				Cancer surveillance
NB37	48	F	NC	TI	–				Fever of unknown origin
NB39	31	F	NC	TI	–				Abdominal pain
NB48	20		NC	AC	–				Rectal bleeding

*CD* Crohn’s disease, UC ulcerative colitis, *NC* normal control, *AU* aphthous ulcer, *SB* small bowel, *LB* large bowel, *REC* rectum, *SIG* sigmoid colon, *DC* descending colon, *AC* ascending colon, *ICV* ileocaecal valve, *CM* caecum, *TI* terminal ileum, *NB* normal (mucosa) biopsy, *F* female, *M* male, *L1* ileal CD, *L2* colonic CD, *L3* Ileocolonic CD, *E3* pancolitis ulcerative colitis (proximal to splenic flexure), *B1* non-stricturing non-penetrating, *B2*, structuring, *B3* penetrating, *p* perianal disease, *N* ‘no’, *Y* ‘yes’ *FHCRC*, family history of colorectal cancer

### Sequence coverage and diversity

**Fig. 1:**
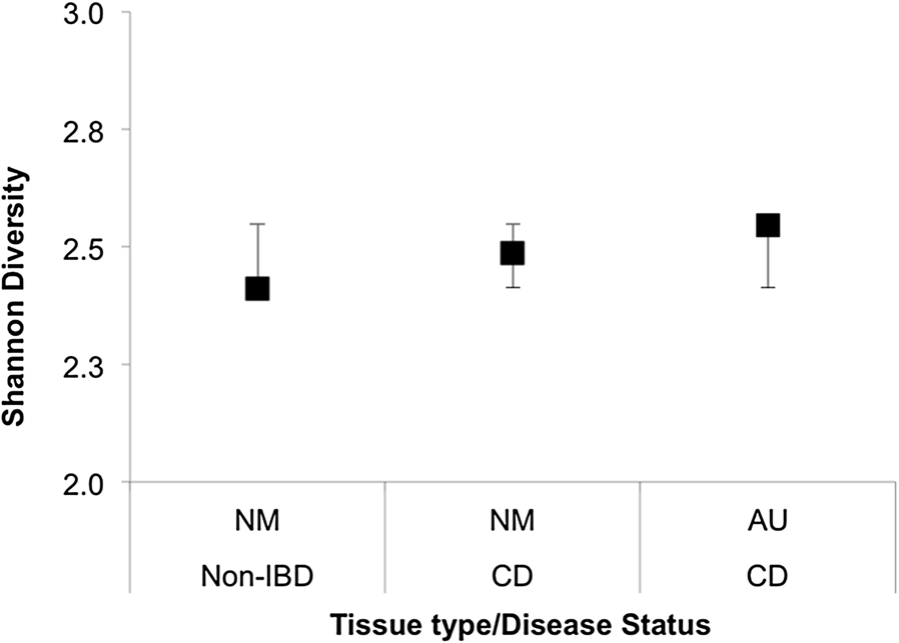
Shannon diversity indices for the microbial communities of Crohn’s disease aphthous ulcers and adjacent mucosa, and healthy control mucosa. Shannon Diversity Indices for the microbial communities of Crohn’s disease aphthous ulcers and macroscopically normal adjacent mucosa, and healthy control (Non-IBD) mucosa. The means and 95% CIs for each sample are depicted. There is no significant difference in the diversity of healthy control mucosa and Crohn’s disease mucosa or aphthous ulcers (ANOVA: F_(1,27)_ = 0.0576, p > F = 0.8125). *NM non-IBD* normal mucosa from healthy controls without inflammatory bowel disease, *NM CD* adjacent normal mucosa from patients with Crohn’s disease, *AU CD* aphthous ulcers from patients with Crohn’s disease

A total of 400,709 raw 16S rRNA gene sequences were generated from all samples, giving an average coverage of 9773 sequences per sample. The diversity of the microbial communities of Crohn’s disease aphthous ulcers and adjacent normal mucosa, and healthy control mucosa, was estimated using the Shannon index. The samples from patients with Crohn’s disease had similar diversity indices to the mucosa from healthy controls (ANOVA: F_(1,27)_ = 0.0576, p > F = 0.8125, Fig. 1).

### Microbial community structure

**Fig. 2:**
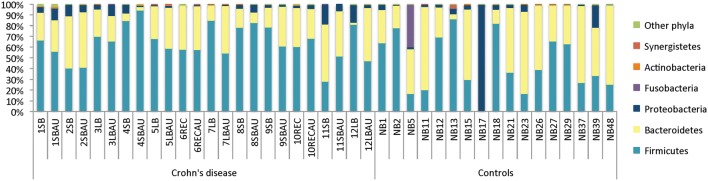
Relative abundance of the dominant bacterial phyla for Crohn’s disease and control tissues. Crohn’s disease tissues: number corresponds to patient; *SB* small bowel, *SBAU* small bowl aphthous ulcer, *LB* large bowel, *LBAU*, large bowel aphthous ulcer, *REC* rectum, *RECAU* rectal aphthous ulcer. Controls: *NB* normal (mucosa) biopsy; number corresponds to patient

We did not observe bacterial imbalance in the majority of biopsies from Crohn’s disease patients, including the aphthous ulcers, based on the relative abundance of the major phyla when compared to control samples (Fig. 2). An analysis of similarities (ANOSIM) revealed that the composition of the microbial communities of aphthous ulcers did not differ significantly from adjacent mucosa from the same patient (p = 0.973), or from mucosa from healthy controls (p = 0.668). The average relative abundance of Firmicutes, which are often decreased in Crohn’s disease [32], was: Crohn’s disease mucosa, 67%; Crohn’s disease aphthous ulcers, 62%; control mucosa, 47%. The average relative abundance of Bacteroidetes was: Crohn’s disease mucosa, 27%; Crohn’s disease aphthous ulcers, 33%; control mucosa, 46%. The average relative abundance of Proteobacteria, which are usually increased in Crohn’s disease [32], was: Crohn’s disease mucosa, 6%; Crohn’s disease aphthous ulcers, 5%; control mucosa, 4%. Control patient NB17 was excluded from the above analyses, because their microbiome comprised 99% *Pseudomonas*, and was therefore a clear outlier.

**Fig. 3:**
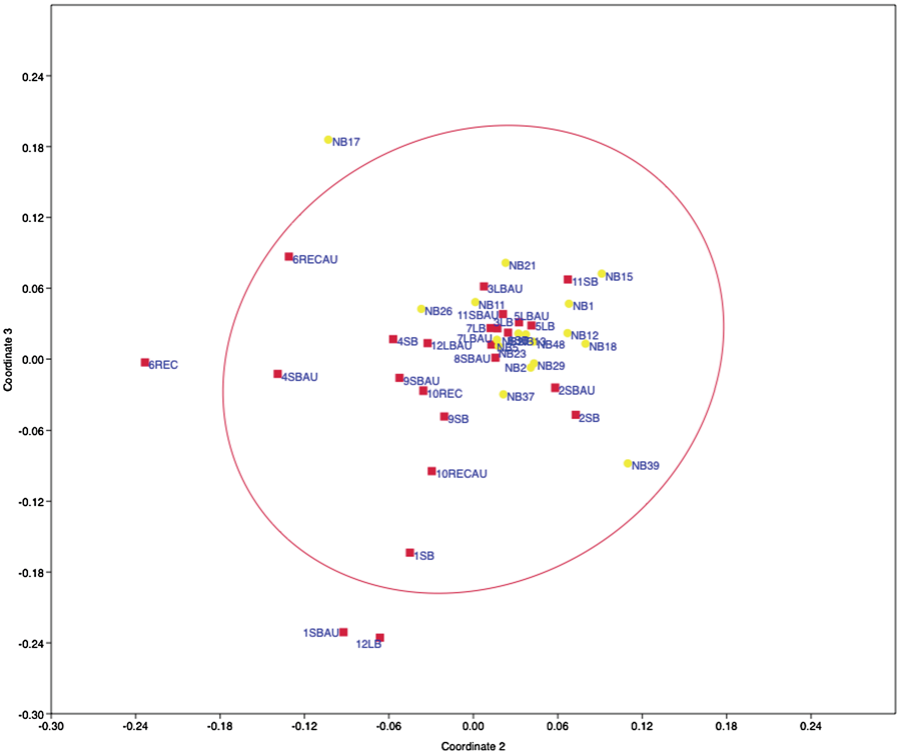
Non-metric dimensional scaling (NMDS) plot of the microbial communities of Crohn’s disease aphthous ulcers and adjacent mucosa, and healthy control mucosa. Non-metric dimensional scaling (NMDS) plot of the microbial 16S rRNA communities of Crohn’s disease aphthous ulcers and adjacent macroscopically normal mucosa (red squares), and mucosa from healthy controls (yellow dots). The Bray-Curtis similarity index was used to determine the distance between two points; sites positioned close together share a greater fraction of their bacterial taxa than two samples further apart. Samples do not cluster according to disease status. Patient numbers precede the tissue type labels: *REC* rectum, *RECAU* rectal aphthous ulcer, *LB* large bowel, *LBAU* large bowel aphthous ulcer, *SB* small bowel, *SBAU* small bowel aphthous ulcer, *NB* normal (control mucosa) biopsy. NMDS stress = 0.40

A distance matrix was calculated for each sample using Oneway PERMANOVA and the Bray–Curtis similarity measure. The matrix was plotted in two dimensions using non-metric dimensional scaling (NMDS) (Fig. 3). The distance between two points in Fig. 3 is directly proportional to the Bray–Curtis similarity value for two samples, such that two samples that are close together have more similar microbial communities than those positioned further apart. The NMDS plot reveals that samples do not cluster by disease status. Samples from the same patient (aphthous ulcers and adjacent normal mucosa) are, on average, more similar to each other than samples from other patients. The distance between aphthous ulcer and adjacent mucosa samples from newly diagnosed patients (6REC/6RECAU; 9SB/9SBAU; 10REC/10RECAU) are similar to that of patients with established disease. Samples from patient 12 with Crohn’s disease, who had been on antibiotics (12LB, 12LBAU), and NB17 (control) were removed from the analysis, as they were clear outliers. The same trends observed above, were also observed using the Jaccard and Theta YC algorithims, also using NMDS to plot the data.

A similarity percentage (Simper) analysis was conducted to determine the percent contribution of each bacterial family to the Bray–Curtis dissimilarity measure, for aphthous ulcers and mucosa from healthy controls (Table 2). Results are reported for families where the average dissimilarity was > 0.1%. The following families had the highest average dissimilarity values: Bacteroidaceae (11.93%); Lachnospiraceae (9.64%); Ruminococcaceae (7.08%); Prevotellaceae (5.08%); Clostridiaceae_1 (2.36%); and Erysipelotrichaceae (2.18%). Of these, Lachnospiraceae, Clostridiaceae_1, Erysipelotrichaceae, and Enterococcaceae had a higher percentage contribution in aphthous ulcers compared to control mucosa.

**Table 2:** Similarity of percentages (SIMPER) results for bacterial community dissimilarity (Bray–Curtis) between bacterial families of aphthous ulcers and control mucosa

Taxon (family)	Avg. Dissim %	Contrib. %	Cumulative %	Mean aphthous	Mean control
Bacteroidaceae	11.93	24.54	24.54	0.31	0.36
Lachnospiraceae	9.64	19.83	44.37	0.33	0.26
Ruminococcaceae	7.08	14.57	58.93	0.13	0.15
Prevotellaceae	5.08	10.45	69.39	0.02	0.09
Clostridiaceae_1	2.36	4.86	74.25	0.05	0.00
Erysipelotrichaceae	2.18	4.49	78.74	0.04	0.03
Enterococcaceae	1.97	4.04	82.78	0.04	0.00
Fusobacteriaceae	1.25	2.58	85.36	0.00	0.03
Peptostreptococcaceae	0.96	1.97	87.33	0.01	0.01
Sutterellaceae	0.83	1.70	89.03	0.02	0.01
Enterobacteriaceae	0.82	1.68	90.71	0.01	0.00
Streptococcaceae	0.66	1.37	92.08	0.00	0.01
Comamonadaceae	0.66	1.36	93.44	0.01	0.01
Veillonellaceae	0.47	0.96	94.40	0.01	0.01
Porphyromonadaceae	0.26	0.54	94.94	0.00	0.00
Hyphomicrobiaceae	0.17	0.34	95.28	0.00	0.00
Clostridiales_Incertae_Sedis_XI	0.17	0.34	95.62	0.00	0.00
Pasteurellaceae	0.14	0.29	95.92	0.00	0.00
Synergistaceae	0.12	0.25	96.16	0.00	0.00
Sphingomonadaceae	0.11	0.22	96.39	0.00	0.00
Desulfovibrionaceae	0.11	0.22	96.61	0.00	0.00
Rikenellaceae	0.11	0.22	96.84	0.00	0.00
Flavobacteriaceae	0.10	0.21	97.05	0.00	0.00

Similarity of percentages (SIMPER) analysis showing the average dissimilarity (%[Avg. Dissim]) of bacterial families represented in aphthous ulcers and control mucosa. Percentage contribution (Contrib. %) is the mean contribution divided by the mean dissimilarity across samples. Only Families with an average dissimilarity > 0.10% are shown

### Presence/absence of bacterial taxa

No bacterial taxa were unique to patients with Crohn’s disease. *Bacteroides* was the only taxon common to all patients and controls, after those on antibiotics were excluded (patients 6 and 12 with Crohn’s disease). *Clostridium cluster XIVa*, and *Lachnospiracea_incertae_sedis* were present in the vast majority of Crohn’s disease and control samples. The relative abundance of *Faecalibacterium* in Crohn’s disease mucosa averaged 14% and was not significantly different to that of control mucosa, which averaged 10% (ANOVA F [1,25] = 0.6869, p > F = 00.415: Wilcoxon [Rank Sums] on untransformed data: χ^2^ = 0.394 p > χ^2^ = 0.5302). There was no significant difference in the relative abundance of *Faecalibacterium* in Crohn’s disease mucosa and aphthous ulcers (Match pairs t test [9_DF_] = 0.957, p > |t| = 0.363). *Faecalibacterium* was not significantly more likely to be detected in control samples: we detected *Faecalibacterium* in 94% of normal controls and 91% of patients with Crohn’s disease (χ^2^ = 0.101 p > χ^2^ = 0.75). Patients 6 and 12 with Crohn’s disease, both on antibiotics, were excluded from these analyses.

## Discussion

This is the first study to assess the microbiome of aphthous ulcers in Crohn’s disease. Our study suggests that the microbiome is not imbalanced in the initial Crohn’s disease lesion, relative to control mucosa. The alpha diversity, and composition of the microbiome of aphthous ulcers and adjacent mucosa from patients with IBD was similar to mucosa from controls. We found no evidence for a reduction in the genus, *Faecalibacterium*, which only contains one species, *Faecalibacterium prausnitzii*, and is commonly found to be decreased in Crohn’s disease. We did not detect an increase in taxa that are usually over-represented in Crohn’s disease mucosa, such as the family Enterobacteriaceae, which includes *E. coli*.

Bacterial community imbalance, or dysbiosis, is a common finding in IBD. Studies often report a decrease in protective groups (such as Lachnospiraceae, *Roseburia*, and *Faecalibacterium*), and a subsequent increase in pathobionts, (such as Proteobacteria, Ruminococcus, and Fusobacterium). Dysbiosis is likely to result from several factors. One study looked at the effects of inflammation, antibiotic exposure, and diet (exclusive enteral nutrition [EEN]) on the gut microbiome of paediatric patients with active Crohn’s disease [33]. They found that each factor independently affected different bacterial taxa in the microbial community. They also showed that dysbiosis decreased with reduced intestinal inflammation, and that the microbiome of patients who responded to anti-TNF therapy and EEN became more similar to healthy controls than that of non-responders. These data support the idea that dysbiosis is a consequence, not cause, of inflammation. We did not control for diet in this study, however we did observe dysbiosis in patients who had consumed antibiotics. The degree or duration of inflammation in the aphthous ulcers may not have been great enough to affect the microenvironment, or to initiate dysbiosis.

A study by Lupp et al. [34]. showed that host-mediated inflammation in response to infection (*Campylobacter jejuni*) and oral administration of dextran sodium sulfate (DSS) leads to dysbiosis in a mouse model. In particular, they observed an expansion in Enterobacteriaceae. A study of the microbiome of a cohort of treatment-naïve new-onset patients with Crohn’s disease, revealed that antibiotic use exaggerates dysbiosis [4]. They also showed that inflammatory conditions were strongly associated with a reduction in species richness and expansion of Enterobacteriaceae, as well as Bacteroidales, and Clostridiales.

The strength of this study was the ability to assess the microbiome of the initial Crohn’s disease lesion (aphthous ulcer), before transmural inflammation and clinical manifestations developed for the first time, or for a new flare. Although our Crohn’s disease cohort was small, we were able to demonstrate that dysbiosis is not a feature of aphthous ulcers. Similar sized cohorts of patients have demonstrated dysbiosis in samples obtained from patients with active Crohn’s disease, including a reduction in Faecalibacterium [35, 36]. If dysbiosis were a feature of the aphthous ulcer microbiome, we would likely have observed it in a number of our Crohn’s disease patients.

It is unclear when dysbiosis of the gut microbiome develops in patients with Crohn’s disease, and if it becomes progressively worse with each disease flare. One study assessed the gut microbiome of unaffected genetically-linked relatives of children with Crohn’s disease. The unaffected relatives had alterations in their gut microbiome in the direction of their relatives with Crohn’s disease, but did not display a distinct dysbiosis [36]. The findings of this study suggest that dysbiosis develops close to disease onset, or as a consequence of the disease process. There is some evidence to suggest that dysbiosis improves over time, but is still evident, in patients with complete mucosal healing or who have responded to treatment [35]. Only one patient with Crohn’s disease in this study (CD11) had previous surgery, all other patients were in remission, or had only mild symptoms. Patients with a long history of mild disease may be less likely to have gut microbiome imbalance.

If dysbiosis does improve in the absence of active disease, then interventions aimed at restoring the gut microbiome may be effective in increasing gut microbial diversity. Reduced alpha diversity could lead to a break-down in the functional redundancy of gut communities, which may exacerbate symptoms. It would be important to administer interventions, such as pro-, pre- and syn-biotics, in the absence of inflammation, as attempts to establish or nourish bacteria that do not cope well in an environment of chronic inflammation and oxidative stress may be futile.

## Conclusions

Our data suggest that dysbiosis is a consequence of the inflammatory disease process, as it was not observed in the initial lesion. We did not detect dysbiosis in the three patients who were newly diagnosed at the time of sampling, nor in patients with more established disease. Longitudinal studies aimed at assessing the gut microbiome before disease onset and throughout successive flares would provide further insight into the nature and development of dysbiosis, but acquiring samples from patients prior to diagnosis remains problematic.

## Methods

### Patient and sample characteristics

Biopsies were collected at the time of colonoscopy by a gastroenterologist, and all diagnoses of IBD were made based on standard criteria: clinical presentation and endoscopic/clinical findings. A total of 41 mucosal biopsies were used for the study: aphthous ulcers (n = 12) and adjacent normal mucosal biopsies (n = 12) from patients with IBD, and normal mucosal biopsies from healthy controls (n = 17). Biopsies from healthy controls were selected from a larger pool, so that they resembled the biopsies from IBD patients with respect to gut region, age, and gender. All samples were stored in RNA*later*^®^ at 4 °C for 24 h, then − 80 °C until required. Table 1 outlines the characteristics for the study participants, including disease status and behaviour, gut location of biopsies, age, gender, and antibiotic usage. Only one patient (CD11) had prior surgery.

### DNA extraction and amplification

Biopsies in RNA*later*^®^ were thawed and DNA was extracted using Qiagen DNeasy Blood and Tissue kits, with the addition of the enzymatic lysis buffer, bead-beating (5000 rpm/30 revs/s for 3 min using a Qiagen TissueLyser II), and RNase A steps, as described in the manufacturer’s protocol. Extraction negative controls were included and were always negative. DNA was amplified using barcoded universal bacterial primers targeting the V1–V3 region of the 16S rRNA gene, and PCR conditions, as previously described [5]. Both positive and negative controls were used for each PCR. DNA was quantified and quality-checked using an Agilent 2100 Bioanalyzer with DNA 1000 chips. Equimolar amounts of the PCR products were combined to make a 500 ng library, which was used as template in the emulsion PCR prior to sequencing on a 454 Genome Sequencer FLX-Titanium system. The sequencing was performed at the Biological Research Facility, ANU, Australia, according to the manufacturer’s instructions (454 Life Sciences, Branford, Connecticut, USA). Signal processing and base calling were performed using 454 Sequencing Software V.2.6 (Roche).

### Sequence processing

Sequence curation and processing were performed in Mothur [37] (v 1.32.1) as previously described [5]. Briefly, sequences were assessed for quality, trimmed of adaptors and barcodes (barcode mismatches allowed, 1 bp; primer mismatches, 2 bp), and chimeras removed using the Uchime code [38]. Sequences were aligned using the Silva database, and taxonomic assignments using the RDP 2012 training sets.

### Statistical analysis

PAST3 was used to generate: non-metric dimensional scaling (NMDS) plots, using the Bray–Curtis similarity measure, using normalized family level taxonomic data as an input; one-way ANOSIM; one-way ANOVA; Simper analysis using the Bray–Curtis measure of similarity. JMP (v.9) was used to conduct one-way ANOVA of Shannon indices between Crohn’s disease and control samples, and to conduct matched pairs *t* test statistics of mean relative abundance figures for individual taxa.
